# Exploration of the molecular targets and mechanisms of suxiao xintong dropping pills for myocardial infarction by network pharmacology method

**DOI:** 10.1042/BSR20204211

**Published:** 2021-08-05

**Authors:** Daqiu Chen, Yanqing Wu, Yixing Chen, Qiaoxing Chen, Xianhua Ye, Shanghua Xu, Shunxiang Luo

**Affiliations:** Department of Cardiology, Affiliated Nanping First Hospital, Fujian Medical University, Nanping 353000, Fujian Province, China

**Keywords:** Chinese Traditional, Medicine, Molecular Docking Simulation, Myocardial Infarction, Pharmacology

## Abstract

**Background:** Suxiao Xintong dropping pills (SXXTDP), a traditional Chinese medicine, is widely applied for treating myocardial infarction (MI). However, its therapy mechanisms are still unclear. Therefore, this research is designed to explore the molecular mechanisms of SXXTDP in treating MI.

**Methods:** The active ingredients of SXXTDP and their corresponding genes of the active ingredients were retrieved from the Traditional Chinese Medicine Systems Pharmacology (TCMSP) database. MI-related genes were identified via analyzing the expression profiling data (accession number: GSE97320). Gene Ontology (GO) and Kyoto Encyclopedia of Genes and Genomes (KEGG) pathway enrichment analysis were performed to study the shared genes of drug and disease. Through protein–protein interaction (PPI) network and the Cytoscape plugin cytoHubba, the hub genes were screened out. The compounds and hub targets binding were simulated through molecular docking method.

**Results:** We obtained 21 active compounds and 253 corresponding target genes from TCMSP database. 1833 MI-related genes were identified according to *P*<0.05 and |log_2_FC| ≥ 0.5. 27 overlapping genes between drug and disease were acquired. GO analysis indicated that overlapping genes were mainly enriched in MAP kinase activity and antioxidant activity. KEGG analysis indicated that overlapping genes were mainly enriched in IL-17 signaling pathway and TNF signaling pathway. We obtained 10 hub genes via cytoHubba plugin. Six of the 10 hub genes, including *PTGS2, MAPK14, MMP9, MAPK1, NFKBIA, and CASP8*, were acted on molecular docking verification with their corresponding compounds of SXXTDP.

**Conclusion:** SXXTDP may exert cardioprotection effect through regulating multiple targets and multiple pathways in MI.

## Introduction

Myocardial infarction (MI) is a class of cardiovascular diseases that possess high mortality all over the world [1]. Percutaneous coronary intervention (PCI) and thrombolytic therapy are considered as the best treatment strategies to rescue the endangered myocardium in MI. Nevertheless, the ischemic but still viable cardiomyocytes don’t always be saved after reperfusion. Inflammatory and oxidative stress responses will lead to further cardiomyocyte necrosis after MI. Hence, it is clinically significant to seek for an approach to inhibit inflammatory and oxidative stress responses after MI, which will protect the ischemic but still viable cardiomyocytes.

Some Traditional Chinese Medicine (TCM) can improve blood circulation. Therefore, TCM has been employed to treat ischemia cardiovascular diseases in China for a long time. SXXTDP comprising Chuanxiong (Rhizoma Chuanxiong), Mudanpi (Cortex Moutan), and Bingpian (Borneolum Syntheticum). SXXTDP has broad pharmacological activities, including anti-inflammation, anti-oxidant, anti-arrhythmic activity, and so on [2,3]. As is known to all, western medicine is single target therapy, which sometimes can not exert optimal effects on the treatment of complicated diseases. In contrast, TCM has the characteristics of multiple targets and multiple pathways in the treatment of diseases. At present, it remains unclear what are the potential molecular mechanisms of SXXTDP in treating MI.

Network pharmacology is an effective method to discover novel drugs and mechanisms. So far, this method has been successfully used to clarify the multi-target regulation of TCM in treating diseases. For example, Li et al. explored the potential mechanisms of Tongxinluo capsule in the treatment of coronary heart disease [4]. Yu et al. found that the mechanisms of XiaoLuoWan in treating uterine fibroids by the network pharmacology method [5]. The ultimate goal is to raise the treatment effect, reduce the side effect, and accelerate the development of novel drugs.

In the present study, we find the active ingredients and potential molecular mechanisms of SXXTDP in treating MI by utilizing network pharmacology method. These findings indicate that SXXTDP is effective in anti-inflammation, anti-oxidant, anti-arrhythmic activity, and anti-apoptosis, and thus can be applied to the treatment of MI.

## Methods

### Identification of the active compounds of SXXTDP and corresponding to target genes

All chemical constituents of SXXTDP were retrieved from the Traditional Chinese Medicine Systems Pharmacology (TCMSP) database (https://tcmspw.com/tcmsp.php) [6]. We selected the oral bioavailability (OB) ≥30% and drug-likeness (DL) ≥0.18 as the screening criteria [7,8]. The target genes corresponding to active compounds of SXXTDP were screened out from TCMSP database. Obtained target genes were imported into UniProt (https://www.uniprot.org/) [9] to search for their information, including the gene symbol, gene ID, and so on [10].

### Identification of target genes related to MI

The expression profiling data of GSE97320 and annotation information of microarray platform GPL570 (Affymetrix Human Genome U133 Plus 2.0 Array) were acquired from the GEO database (https://www.ncbi.nlm.nih.gov/geo/), including 3 samples from healthy individuals and 3 MI samples. On the basis of the annotation information of platform GPL570, probe IDs were converted to the corresponding genes. According to *P*<0.05 and |log_2_FC| ≥ 0.5, differential expression genes (DEGs) between healthy individuals and MI were obtained by using the package limma of R language [11] and were visualized by a volcano plot.

### Acquisition of overlapping genes between drug and disease, construction of a drug-compound-target genes network

Using the Perl language, we acquired the overlapping target genes of drug and disease. By using Cytoscape 3.8.0 software [12], ‘drug-compound-target’ network was established. In the network diagram, the nodes of triangle and ellipse represent active compounds of drug and target genes, respectively, and they are connected by edges.

### Establishing PPI network of overlapping genes and selection of hub genes

The overlapping genes of drug and disease were imported into the STRING database (https://string-db.org/) [13] and a protein–protein interaction (PPI) network was constructed. The screening conditions were the species as “Homo sapiens” and the combined score >0.4. In the PPI diagram, each node represents a gene and the nodes are connected by lines. By using the Cytoscape plugin cytoHubba [14], the key genes were identified by Maximal Clique Centrality (MCC) method.

### GO enrichment analysis

Gene Ontology (GO) analysis is an important method that describes the features of candidate targets. By using Clusterprofiler package [15], a bioconductor package [16], the shared target genes of drug and disease were analyzed with the GO enrichment analysis tool. The screening criteria was *P*<0.05.

### KEGG Pathway Enrichment Analysis

Kyoto Encyclopedia of Genes and Genomes (KEGG) analysis is an important method that describes enrichment of signal pathways of candidate targets. By using Clusterprofiler package, the shared target genes of drug and disease were analyzed with the KEGG pathways analysis tool. The screening criteria was *P*<0.05.

### Molecular docking of the main active ingredients of SXXTDP and core proteins

According to the results of GO analysis and KEGG pathway, we selected the key protein receptor and ligand associated with protein receptor. The 2D chemical structure of small molecular ligands were acquired from PubChem (https://pubchem.ncbi.nlm.gov/) [17]. The 3D chemical structures of small molecular ligands were constructed by using ChemOffice software [18]. The 3D chemical structure of protein receptor was acquired from PDB (http://www.rcsb.org/) [19]. PyMol 2.4.0 software (https://pymol.org.) [20] was used to remove molecular ligands and water molecules of the protein receptor. After installing AutoDock Vina and AutoDockTools-1.5.6 software [21], the format of protein receptor and small molecular ligands was transformed into pdbqt format. The active pocket was subsequently determined. By using Perl language, molecular docking was generated through AutoDock Vina [22]. Based on the binding energy value, the lowest the binding energy value was selected as the docking affinity. Finally, the visualizing 3D structures of molecular ligand and protein receptor bonding were constructed using PyMol software.

## Results

### Identification of the active compounds and corresponding to target genes

According to the screening criteria of the OB≥30% and DL≥0.18, 21 active compounds of SXXTDP were obtained from the TCMSP database, including 3 ingredients of Bingpian, 7 ingredients of Chuanxiong, and 11 ingredients of Mudanpi, as shown in Table 1. From the TCMSP database, we obtained 279 target genes with corresponding to active compounds, including 42 target genes of Chuanxiong and 237 target genes of Mudanpi. 279 full names of genes were converted to gene symbols through Uniprot database. Finally, we obtained 253 target genes after removing duplications. The details of the above data see the supplementary materials (Document 1).

**Table 1:** Compounds in Suxiao Xintong Dropping Pills

MolID	Molecule name	OB(%)	DL
MOL006861	Asiatic acid	41.38	0.71
MOL006862	Bronyl acetate	59.3	0.51
MOL006865	Dipterocarpol	41.71	0.76
MOL001494	Mandenol	42	0.19
MOL002135	Myricanone	40.6	0.51
MOL002140	Perlolyrine	65.95	0.27
MOL002151	Senkyunone	47.66	0.24
MOL002157	Wallichilide	42.31	0.71
MOL000359	Sitosterol	36.91	0.75
MOL000433	FA	68.96	0.71
MOL001925	paeoniflorin_qt	68.18	0.4
MOL000211	Mairin	55.38	0.78
MOL000359	Sitosterol	36.91	0.75
MOL000422	Kaempferol	41.88	0.24
MOL000492	(+)-catechin	54.83	0.24
MOL007003	Benzoyl paeoniflorin	31.14	0.54
MOL007369	4-O-methylpaeoniflorin_qt	67.24	0.43
MOL007374	5-[[5-(4-methoxyphenyl)- 2-furyl]methylene] barbituric acid	43.44	0.3
MOL007382	Mudanpioside-h_qt 2	42.36	0.37
MOL007384	Paeonidanin_qt	65.31	0.35
MOL000098	Quercetin	46.43	0.28

### Identification of target genes related to MI

**Figure 1:**
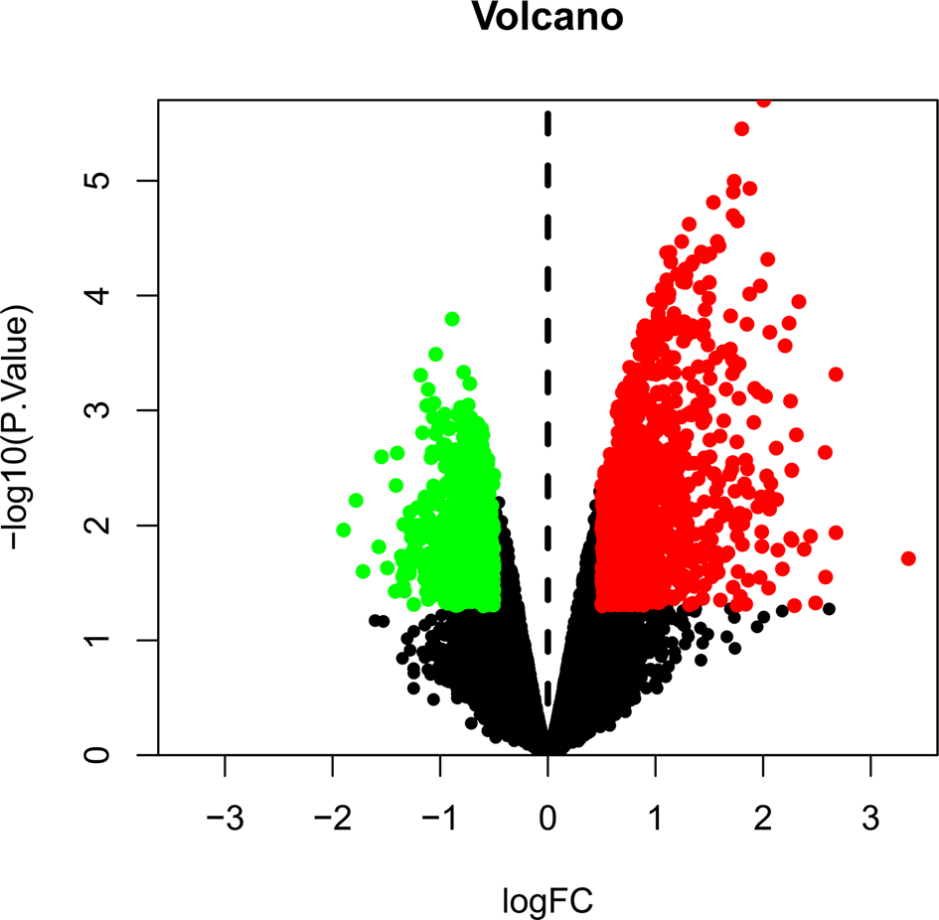
Volcano plot of DEGs of MI The red and green dots represent up-regulated and down-regulated genes, respectively.

According to the criteria of |Log_2_FC| ≥ 0.5 and *P*<0.05, 1833 DEGs, including 1117 up-regulated genes and 716 down-regulated genes, were identified by analyzing the expression profiling data (accession number: GSE97320). A volcano plot of DEGs was shown in Figure 1. The red and green dots represent up-regulated and down-regulated genes, respectively. The details of the above data are shown in the supplementary materials (Document 2).

### Construction of a drug-compound-target genes network

**Figure 2:**
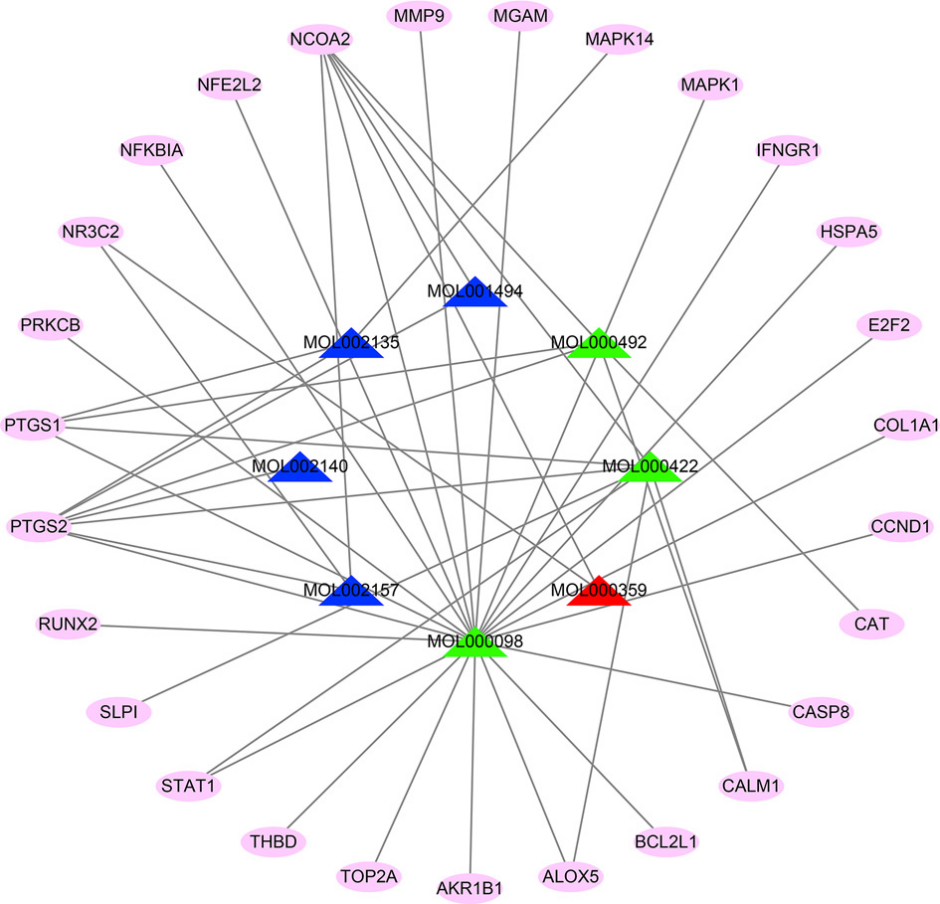
Compound-target network of SXXTDP The blue triangles represent the compounds coming from Chuanxiong. The green triangles represent the compounds coming from Mudanpi. The red triangle represents the compound coming from Chuanxiong and Mudanpi. The node of pink ellipse represents target genes.

About 27 overlapping genes related with MI and SXXTDP were obtained through the Perl language, including *PTGS2, NCOA2, PTGS1, MAPK14, NR3C2, CALM1, STAT1, ALOX5, SLPI, CAT, AKR1B1, CCND1, BCL2L1, MMP9, MAPK1, NFKBIA, CASP8, HSPA5, PRKCB, MGAM, THBD, COL1A1, IFNGR1, TOP2A, NFE2L2, RUNX2*, and *E2F2*. Next, the network of ‘drug-compounds-targets’ was constructed by utilizing Cytoscape 3.8.0 software, as shown in Figure 2. This network explained that compounds of SXXTDP could interfere with MI via binding multiple target genes.

### Establishing PPI network of overlapping genes and selection of hub genes

**Figure 3:**
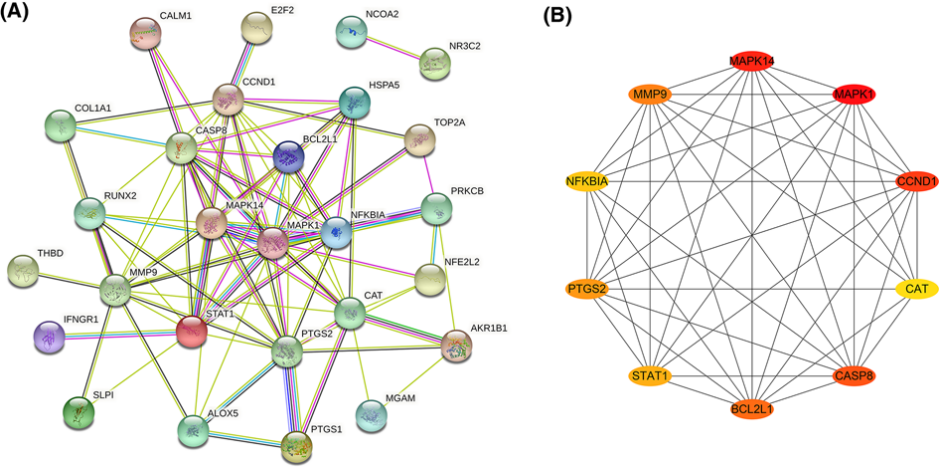
Protein-protein interaction network (**A**) PPI network of MI. Each node represents one target gene. The lines of different colors represent different sources of the evidence for protein–protein interaction. (**B**) The core genes are sorted by MCC method. The ellipse nodes represent genes. The more lines are there in the network, the more important the genes are.

About 27 overlapping genes associated with disease and drug were inputted into STRING database. We obtained a PPI network after selecting “Homo sapiens” and the medium confidence>0.4, as shown in Figure 3A. There were 27 nodes and 89 edges in this network. Using the Cytoscape plugin cytoHubba, we obtained the top 10 key genes by MCC method, including *MAPK1, MAPK14, CCND1, CASP8, BCL2L1, MMP9, PTGS2, STAT1, NFKBIA*, and *CAT*, as shown in Figure 3B.

### GO enrichment analysis

**Figure 4:**
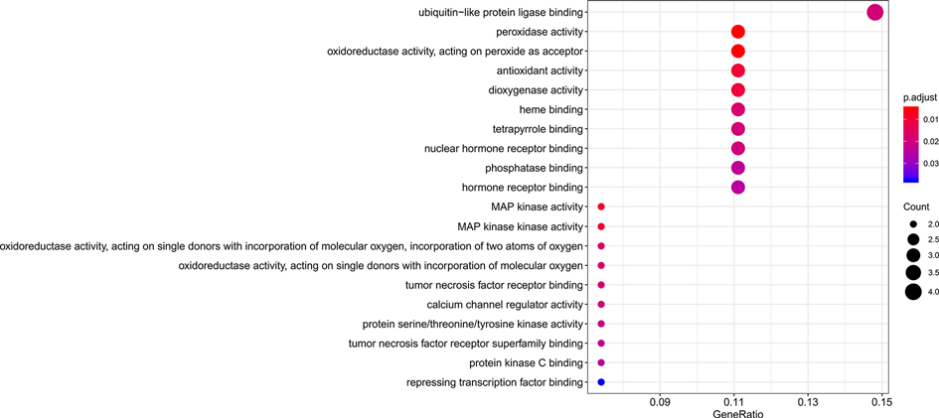
Compound-target network of SXXTDP On the basis of *P* value, the top 20 GO functional terms were selected. The color represents *P* value and the size of the dot represents the number of genes.

In order to further analyze the function of 27 overlapping genes, GO enrichment analysis was conducted by the clusterProfiler package in R language. The top 20 GO enrichment terms (adjusted, *P*<0.05) were presented in Figure 4. The top 10 GO terms were listed in Table 2.

**Table 2:** The top 10 GO terms of 27 overlapping genes

GO terms	Description	Adjusted *P*-value	Corresponding genes
GO:0004601	Peroxidase activity	0.00499534	*PTGS2, PTGS1, CAT*
GO:0016684	Oxidoreductase activity, acting on peroxide as acceptor	0.00499534	*PTGS2, PTGS1, CAT*
GO:0004707	MAP kinase activity	0.009969697	*MAPK14, MAPK1*
GO:0004708	MAP kinase kinase activity	0.009969697	*MAPK14, MAPK1*
GO:0016209	Antioxidant activity	0.009969697	*PTGS2, PTGS1, CAT*
GO:0051213	Dioxygenase activity	0.010516935	*PTGS2, PTGS1, ALOX5*
GO:0016702	Oxidoreductase activity, acting on single donors with incorporation of molecular oxygen, incorporation of two atoms of oxygen	0.017094371	*PTGS2, ALOX5*
GO:0016701	Oxidoreductase activity, acting on single donors with incorporation of molecular oxygen	0.017094371	*PTGS2, ALOX5*
GO:0020037	Heme binding	0.018048512	*PTGS2, PTGS1, CAT*
GO:0005164	Tumor necrosis factor receptor binding	0.018048512	*STAT1, CASP8*

### KEGG pathway enrichment analysis

**Figure 5:**
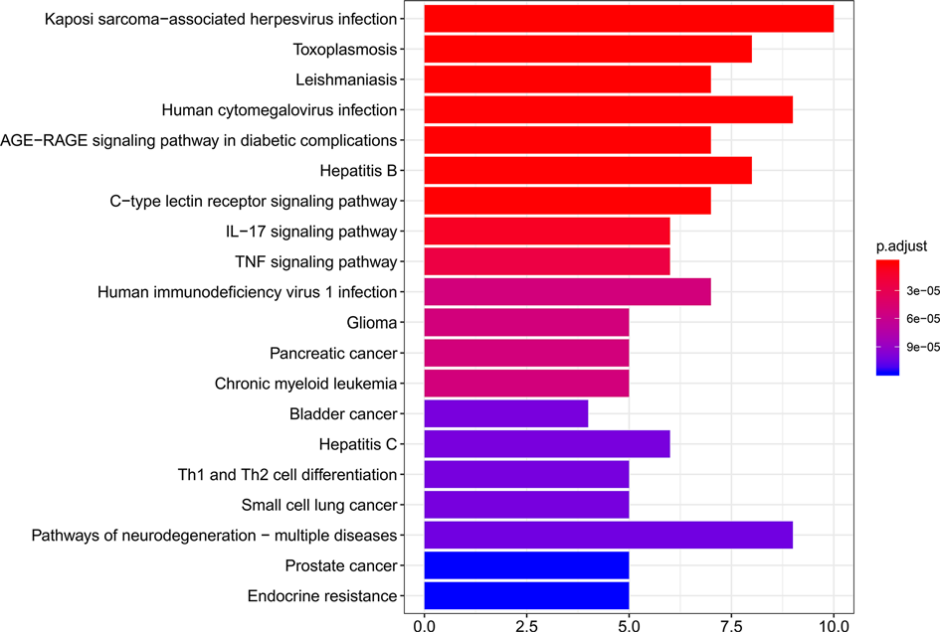
Compound-target network of SXXTDP On the basis of *P* value, the top 20 pathways are shown. The color represents *P* value and the length of the column represents the number of genes.

KEGG enrichment analysis was conducted by the clusterProfiler package in R language. The top 20 KEGG enrichment terms (adjusted, *P*<0.05) were presented in Figure 5. The top 10 KEGG pathways were listed in Table 3.

**Table 3:** The top 10 KEGG pathways of 27 overlapping genes

ID	Pathway name	Adjusted *P*<0.05	Corresponding genes
hsa05167	Kaposi sarcoma-associated herpesvirus infection	3.48E-08	*PTGS2, MAPK14, CALM1, STAT1, CCND1, MAPK1, NFKBIA, CASP8, IFNGR1, E2F2*
hsa05145	Toxoplasmosis	1.27E-07	*MAPK14, STAT1, ALOX5, BCL2L1, MAPK1, NFKBIA, CASP8, IFNGR1*
hsa05140	Leishmaniasis	1.92E-07	*PTGS2, MAPK14, STAT1, MAPK1, NFKBIA, PRKCB, IFNGR1*
hsa05163	Human cytomegalovirus infection	6.88E-07	*PTGS2, MAPK14, CALM1, CCND1, MAPK1, NFKBIA, CASP8, PRKCB, E2F2*
hsa04933	AGE-RAGE signaling pathway in diabetic complications	6.88E-07	*MAPK14, STAT1, CCND1, MAPK1, PRKCB, THBD, COL1A1*
hsa05161	Hepatitis B	6.88E-07	*MAPK14, STAT1, MMP9, MAPK1, NFKBIA, CASP8, PRKCB, E2F2*
hsa04625	C-type lectin receptor signaling pathway	6.88E-07	*PTGS2, MAPK14, CALM1, STAT1, MAPK1, NFKBIA, CASP8*
hsa04657	IL-17 signaling pathway	9.38E-06	*PTGS2, MAPK14, MMP9, MAPK1, NFKBIA, CASP8*
hsa04668	TNF signaling pathway	2.36E-05	*PTGS2, MAPK14, MMP9, MAPK1, NFKBIA, CASP8*
hsa05170	Human immunodeficiency virus 1 infection	5.18E-05	*MAPK14, CALM1, BCL2L1, MAPK1, NFKBIA, CASP8, PRKCB*

### Molecular docking of the main active ingredients of SXXTDP and core proteins

**Figure 6:**
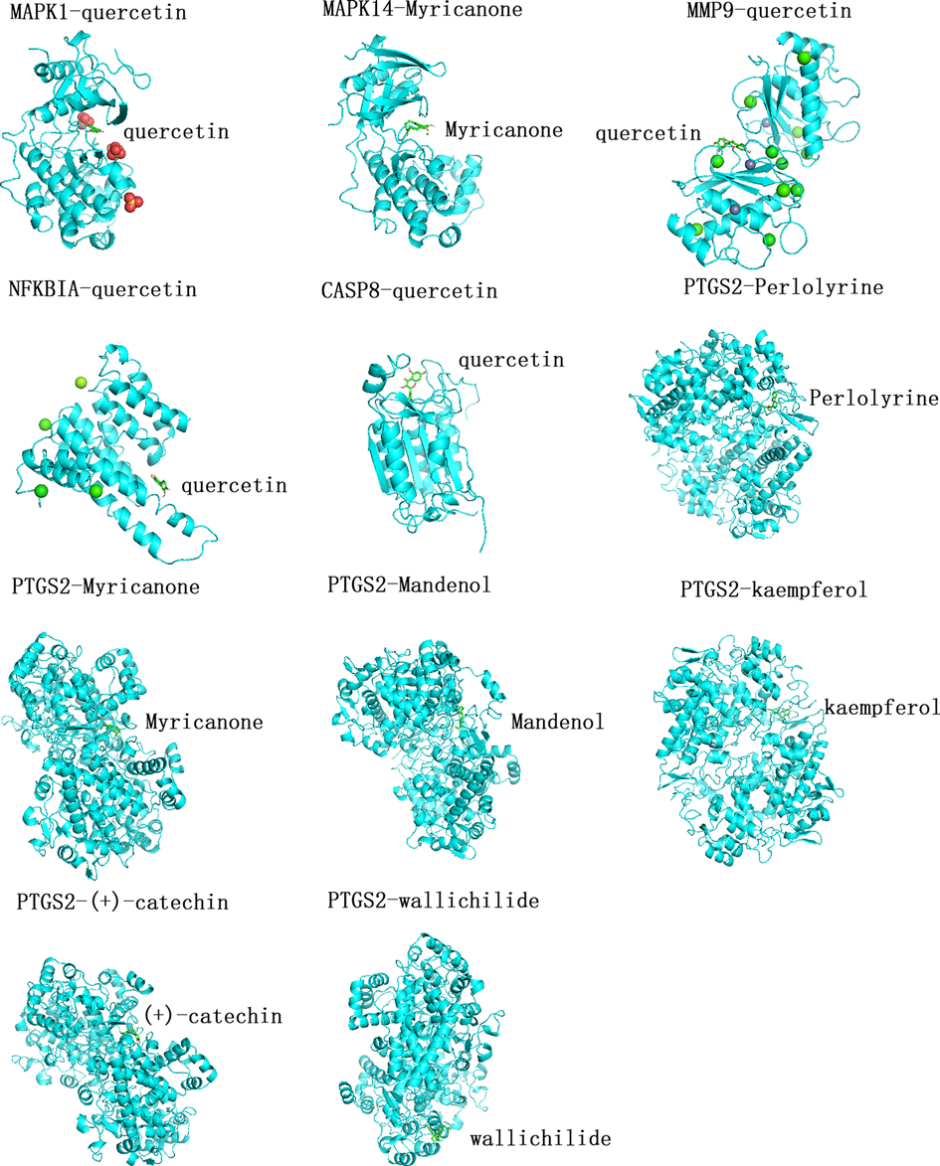
Molecular docking models Molecular model of the ingredient is in the binding pocket of the protein, which is displayed via 3D-map technology. The ingredients are displayed in a ringlike structure colored green.

**Figure 7:**
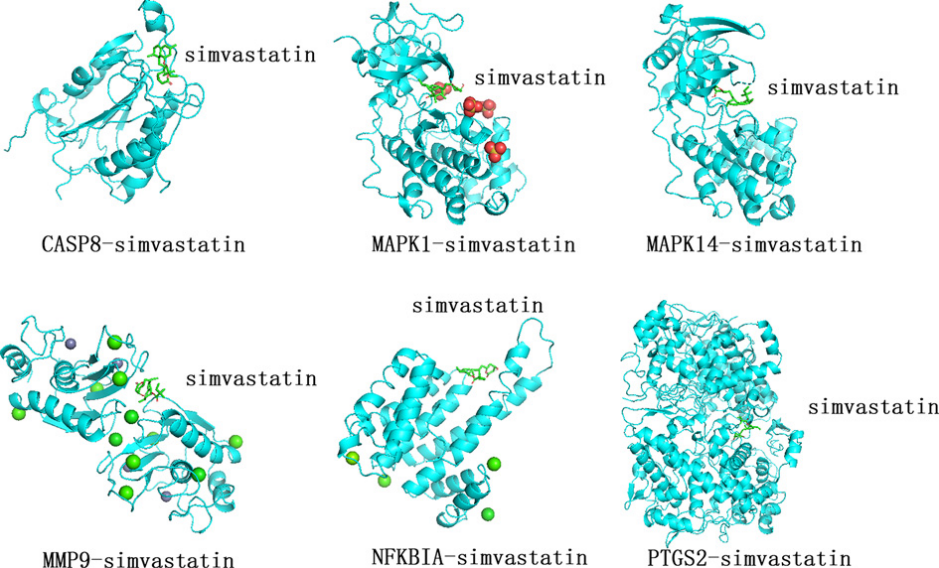
Molecular docking models Molecular model of simvastatin is in the binding pocket of the protein, which is displayed via 3D-map technology. Simvastatin is displayed in a ringlike structure colored green.

Using molecular docking approach, we verified the binding sites of the target genes and their corresponding compounds of SXXTDP. According to IL-17 signaling pathway and TNF signaling pathway from the results of KEGG pathway enrichment analysis, we determined six key genes, including *PTGS2, MAPK14, MMP9, MAPK1, NFKBIA*, and *CASP8.* We identified that quercetin was the ligand of *MMP9, MAPK1, NFKBIA*, and *CASP8* protein receptors. The 2D structure of quercetin was obtained by using the PubChem database, and then the 2D structure of quercetin was converted to 3D structure through ChemOffice software. We acquired the 3D chemical structures of *MMP9, MAPK1, NFKBIA*, and *CASP8* protein receptors from PDB. The water molecules and molecule ligands of *MMP9, MAPK1, NFKBIA*, and *CASP8* protein receptors were removed by utilizing PyMol 2.4.0 software. We obtained the visualizing 3D structures of quercetin, *MMP9, MAPK1, NFKBIA*, and *CASP8* protein receptors bonding by using AutoDockTools and AutoDock Vina. The greater the absolute value of the docking affinity, the more powerful the binding ability between the active site of the protein receptor and the compound. In accordance with the above method, MAPK14-Myricanone, PTGS2-Perlolyrine, PTGS2-Myricanone, PTGS2-Mandenol, PTGS2-kaempferol, PTGS2-(+)-catechin, and PTGS2-wallichilide were verified by molecular docking, as shown in Figure 6. A total of eleven pairs entered into the docking simulation, including MAPK1-quercetin docking (-8.4 kcal/mol), MAPK14-Myricanone docking (-7.2 kcal/mol), MMP9-quercetin docking (-7.9 kcal/mol), NFKBIA-quercetin docking (-7.7 kcal/mol), CASP8-quercetin docking (-7.7 kcal/mol), PTGS2-Perlolyrine docking (-8.0 kcal/mol), PTGS2-Myricanone docking (-7.6 kcal/mol), PTGS2-Mandenol docking (-6.1 kcal/mol), PTGS2-kaempferol docking (-9.3 kcal/mol), PTGS2-(+)-catechin docking (-8.5 kcal/mol), and PTGS2-wallichilide docking (-6.2 kcal/mol), as shown in Table 4. From the docking result, most binding complexes possessed high binding affinity. Next, simvastatin was set as a positive control. The 2D structure of simvastatin was obtained by using the PubChem database, and then the 2D structure of simvastatin was converted to 3D structure through ChemOffice software. We docked simvastatin with the above 6 key genes and found simvastatin had certain binding activity with MAPK1, MAPK14, MMP9, NFKBIA, CASP8, and PTGS2 (see Table 5). MAPK1-simvastatin, MAPK14-simvastatin, MMP9-simvastatin, NFKBIA-simvastatin, CAPS8-simvastatin, and PTGS2-simvastatin were verified by molecular docking, as shown in Figure 7.

**Table 4:** Results of the molecular docking of the six core genes with compounds of SXXTDP

Number	Core genes	PDB ID	Compound	Docking affinity (kcal/mol)
1	MAPK1	5lck	Quercetin	-8.4
2	MAPK14	2qd9	Myricanone	-7.2
3	MMP9	2ow1	Quercetin	-7.9
4	NFKBIA	6y1j	Quercetin	-7.7
5	CASP8	3kjq	Quercetin	-7.7
6	PTGS2	5f19	Perlolyrine	-8.0
			Myricanone	-7.6
			Mandenol	-6.1
			Kaempferol	-9.3
			(+)-catechin	-8.5
			Wallichilide	-6.2

**Table 5:** Results of the molecular docking of the six core genes with simvastatin

Number	Core genes	PDB ID	Compound	Docking affinity (kcal/mol)
1	MAPK1	5lck	Simvastatin	-8.2
2	MAPK14	2qd9	Simvastatin	-8.0
3	MMP9	2ow1	Simvastatin	-7.3
4	NFKBIA	6y1j	Simvastatin	-8.1
5	CASP8	3kjq	Simvastatin	-7.4
6	PTGS2	5f19	Simvastatin	-9.2

## Discussion

The leading causes of death in rural and urban residents were cardiovascular diseases according to China Cardiovascular Diseases Report 2017. Despite advances in medical technology in recent years, myocardial infarction (MI) continues to be regarded as a pivotal risk factor of death [23]. TCM has been employed to treat ischemia cardiovascular diseases in China for a long time. Therefore, it is imperative to develop novel drugs for MI treatment. Due to the multi-target treatment effects of TCM, it can serve as a significant repository to develop drugs for the treatment of MI.

The present study used network pharmacology and molecular docking simulation to research the molecular mechanisms of SXXTDP in the treatment of MI. It was found that SXXTDP exerted a potential role in treating MI through regulating multiple target genes, including *MAPK1, MAPK14, CCND1, CASP8, BCL2L1, MMP9, PTGS2, STAT1, NFKBIA*, and *CAT*. SXXTDP is comprised of Chuanxiong, Mudanpi, and Bingpian. Those ingredients have broad pharmacological activities, including anti-inflammation, anti-oxidant, anti-arrhythmic activity, and so on [2,3]. The active compounds and target genes of SXXTDP were predicted by using the TCMSP database. We obtained 21 active ingredients of SXXTDP and 253 corresponding target genes in total. About 1833 MI-related genes were identified according to *P*<0.05 and |log_2_FC| ≥ 0.5. A total of 27 overlapping genes between drug and disease were acquired. GO analysis indicated that overlapping genes were mainly enriched in MAP kinase activity, antioxidant activity. KEGG analysis indicated that overlapping genes were mainly enriched in IL-17 signaling pathway and TNF signaling pathway. We obtained 10 hub genes via cytoHubba plugin. Six of the 10 hub genes, including *PTGS2, MAPK14, MMP9, MAPK1, NFKBIA*, and *CASP8*, were acted on molecular docking verification with their corresponding compounds of SXXTDP. Study finds that IL-17 signaling pathway is involved in immune responses [24]. Inhibition of IL-17 signaling pathway can improve immune response balance and attain cardioprotection in rats with heart failure [25]. MIR-324/SOCS3 axis can regulate TNF signaling pathway and further improve the hypoxia/reoxygenation-induced myocardial injury [26]. Study finds that d-Limonene alleviates myocardial infarction injury via antioxidant effect [27]. Rosuvastatin can improve cardiac function via reducing p38 MAP kinase activity in rats after myocardial infarction [28].

Mitogen-activated protein kinase 1 (also known as MAPK1) and mitogen-activated protein kinase 14 (also known as MAPK14) are two members of MAP kinase family. MAP kinases are involved in many cellular processes such as transcription regulation and proliferation. Cardiomyocyte-specific deletion of the Mapk14 and Mapk11 genes reduce myocardial cells apoptosis and increase cardiomyocytes proliferation via inactivation of p38 activity [29]. Matrix metallopeptidase 9 (also named as MMP9) belongs to proteins of the matrix metalloproteinase family, which participates the breakdown of extracellular matrix. Many studies report that MMPs exert a main role in atherosclerotic plaque disruption and result in myocardial infarction. Study finds that Kai-Xin-San can protect cardiomyocytes effect via regulating MMPs [30]. NFKB inhibitor alpha (also named as NFKBIA) participates in inflammatory responses. Study finds that blocking NFKBIA-mediated NF-κB signalling pathway can protect against myocardial infarction in mice [31]. Caspase 8 (CASP8) is one of the members of the cysteine-aspartic acid protease (caspase) family, which plays an important role in the execution phase of cell apoptosis. Study finds that Ebselen can inhibit myocardial apoptosis by reducing the expression of caspase-8 and caspase-3 [32]. Prostaglandin-endoperoxide synthase 2 (also named as PTGS2) acts both as a peroxidase and as a dioxygenase. Study finds that miR-26b can improve myocardial remodel and reduce the inflammatory response in mice with myocardial infarction via inhibiting PTGS2 to activate the MAPK pathway [33].

Quercetin, a flavonoid, has special biological functions, including antioxidant, anti-inflammatory, anti-platelet aggregation, and so on [34]. A study reports that quercetin has evidently antioxidant, anti-apoptotic, and anti-inflammatory effects on rat with MI and can protect against cardiomyocytes injury [35]. Myricanone has anti-oxidant and anti-inflammatory [36]. Perlolyrine has antiproliferative activities [37], which may improve myocardial remodel. Kampeferol, a dietary flavonoid, has the characteristic of antioxidant activities, anti-inflammatory, and anti-apoptotic. Study finds that Kampeferol protects against apoptosis and oxidative stress damage of myocardial cells in rats with isoproterenol-induced cardiac toxicity [38]. (+)-catechin, a bioactive polyphenol, has antioxidant property. Study reports that catechin can alleviate hypoxia/reoxygenation-induced cardiomyocytes apoptosis by down-regulating lncRNA MIAT [39]. At present, there are very limited researches about Wallichilide and Mandenol.

Simvastatin, HMG-CoA reductase inhibitor, is effective in antioxidant activity [40], anti-apoptosis [41], and anti-inflammatory effect [42]. Simvastatin can improve reparative fibrosis post-myocardial infarction [43] and cardiac function after myocardial infarction and decrease myocardial apoptosis [44]. Therefore, simvastatin was set as a positive control and its values were compared with the active ingredients of SXXTDP. Interestingly, the affinity of key genes binding with quercetin, myricanone, and kaempferol is similar to that of simvastatin. Having referred to lots of documents, we found that simvastatin inhibited the expression of CASP8 [43], MAPK1 [45], MMP9 [46], and PTGS2 [47]. These results indicated that MAPK1, MAPK14, MMP9, NFKBIA, CASP8, and PTGS2 may be the key targets for the pharmacological action of SXXTDP in treating myocardial infarction.

The results of this research showed that the active ingredients of SXXTDP played an active role in anti-apoptosis, anti-oxidation, anti-inflammation, and improvement of cardiac remodeling roles. According to the molecular docking simulation, we found that MAPK1-quercetin, MMP9-quercetin, NFKBIA-quercetin, CASP8-quercetin, MAPK14-Myricanone, PTGS2-Perlolyrine, PTGS2-Myricanone, PTGS2-kaempferol, and PTGS2-catechin might exert important roles in treating MI.

## Conclusion

The present study reveals that SXXTDP exert cardioprotection effect via regulating multiple targets and multiple pathways in MI. SXXTDP may be a promising drug in treating MI. Meanwhile, our study will provide a scientific basis for the further lab studies.

## Supplementary Material

Supplementary Materials

## Data Availability

The data used to support the findings of this study are included in the article and Supplementary Materials.

## Abbreviations

DEG: differential expression genes
DL: drug-likeness
GO: Gene Ontology
KEGG: Kyoto Encyclopedia of Genes and Genomes
MI: myocardial infarction
OB: oral bioavailability
PCI: percutaneous coronary intervention
PPI: protein–protein interaction
TCM: Traditional Chinese Medicine
TCMSP: Traditional Chinese Medicine System Pharmacology
